# Analysis and synthesis of a growing network model generating dense scale-free networks via category theory

**DOI:** 10.1038/s41598-020-79318-7

**Published:** 2020-12-18

**Authors:** Taichi Haruna, Yukio-Pegio Gunji

**Affiliations:** 1 Department of Information and Sciences, School of Arts and Sciences, Tokyo Woman’s Christian University, 2-6-1 Zempukuji, Suginami-ku, Tokyo 167-8585 Japan; 2 Department of Intermedia Art and Science, School of Fundamental Science and Technology, Waseda University, 3-4-1 Ohkubo, Shinjuku-ku, Tokyo 169-8555 Japan

**Keywords:** Complex networks, Applied mathematics

## Abstract

We propose a growing network model that can generate dense scale-free networks with an almost neutral degree−degree correlation and a negative scaling of local clustering coefficient. The model is obtained by modifying an existing model in the literature that can also generate dense scale-free networks but with a different higher-order network structure. The modification is mediated by category theory. Category theory can identify a duality structure hidden in the previous model. The proposed model is built so that the identified duality is preserved. This work is a novel application of category theory for designing a network model focusing on a universal algebraic structure.

## Introduction

Networks whose degree distribution $p_k$ follows a power-law $p_k \sim k^{-\gamma }$ are called scale-free networks and have been playing a fundamental role in understanding real-world networks in this two decades^1,2^. Here, $p_k$ is the fraction of the nodes of degree *k* in a given network. The Barabási–Albert model (BA model) is one of the most familiar growing network model that can generate scale-free networks with exponent $\gamma =3$^3^. It has been shown that variants of the BA model can generate scale-free networks with arbitrary exponent in the range $\gamma >2$^4^.

When $\gamma >2$, the generated networks by a growing network model are sparse in the sense that the average degree does not diverge as the networks grow. On the other hand, it has been reported that dense scale-free networks are sometimes observed in online social networks and other real-world networks^5,6^. Here, we call a network dense when its average degree diverges as it grows^7,8^. In order for scale-free networks generated by a growing mechanism to be dense, the following two requirements need to be satisfied: First, the power-law exponent $\gamma$ should be less than or equal to 2 in order for the average degree to diverge. Second, there should exist a cutoff for the power-law regime due to a constraint resulting from the maximum degree in a network^9^. So far, only a few growing network models for generating dense scale-free networks with specific values of $\gamma$ have been known (For example, $\gamma =2$^10^ and $\gamma =\frac{3}{2}$^5^).

Recently, the authors proposed a growing network model that can generate dense scale-free networks with arbitrary exponents in the range $\gamma >1$^11^ by modifying a copying model^12^. However, the generated networks by this model have a rather distorted higher-order network structure: They have a strong positive degree−degree correlation, namely, the degree correlation function $k_{\mathrm{nn}}(k)$^13^ increases linearly as the degree of nodes *k* increases on average, and the local clustering coefficient *C*(*k*)^14^ has a tendency that is rarely observed in real-world networks, namely, *C*(*k*) increases as *k* increases on average^11^.

In this paper, by modifying our previous model^11^, we propose a growing network model that can generate dense scale-free networks with a different higher-order structure such as an almost neutral degree−degree correlation and a negative scaling of local clustering coefficient. We show that the proposed model can generate dense scale-free networks in which $k_{\mathrm{nn}}(k)$ is an almost constant function of *k* on average and *C*(*k*) is a decreasing function of *k* on average. In particular, the latter is a hallmark of a hierarchical structure or a modular structure, and frequently observed in real-world networks^14,15^.

The modification of our previous model relies on category theory^16^. Category theory is a kind of abstract algebra that has been used to extract common mathematical structures in different fields of mathematics and transfer a theory in one field to another field^17^. Recently, it has been suggested that category theory can also have effective applications in different fields of science^18^: control theory^19,20^, electrical circuits^21^, reaction networks^22^, databases^23^, resource theory^24^, dynamical systems^25^, machine learning^26,27^, complex systems design^28^, and so on. We use category theory for identifying a hidden duality structure in our previous model of growing networks and making use of it for building a new model. In order to obtain the new model, we modify the previous model so that the duality structure is preserved. Here, we only use a small part of category theory. In particular, we only need the notions of preordered sets and the Galois connections. These materials are reviewed in "Methods".

## Background

Recall that the algorithm of the BA model consists of two steps: growth and preferential attachment (PA)^3^. In the growth step, a new node enters into an existing network. The degree of the new node is given as a fixed value *m*. Then, in the PA step, each existing node acquires a new link to the new node with probability proportional to its degree. The network grows as these two steps are repeated indefinitely.

The copying model focused on in our previous work^11^ was originally proposed as a model of evolution of protein-protein interaction networks driven by gene duplications and mutations^12^. The mechanism of PA is not directly implemented in the model algorithm. However, it naturally gives rise to PA. The copying model replaces the above two steps in the BA model by the copying step and the divergence step, respectively. In the copying step, a new node is produced by copying a randomly chosen existing node together with links emanating from it. In the divergence step, each link from the new node is deleted with a given probability $0<p<1$. PA follows from the copying step because the higher the degree of a node is, the higher the probability that it is reached from a randomly chosen node is. It is known that the generated networks by the copying model have a power-law degree distribution $p_k \sim k^{-\gamma }$ with $2 \le \gamma \le 3$ when $p \le \frac{1}{2}$, and they are dense but not scale-free for $p>\frac{1}{2}$^12^.

In our previous model^11^, only the degree of a randomly chosen node is copied when creating a new node. The copied degree of the node is interpreted as an evaluation value of the ability to form link (‘popularity’). After multiplying a conversion coefficient $\delta >0$ from the degree (a result of link formation) to the ability (a cause of link formation), the obtained value is called the virtual degree of the new node. The targets of links from the new node are determined by a weak form of PA called ordinal preferential attachment (OPA). In OPA, the new node connects to randomly chosen existing nodes whose evaluation value of the ability to form links is greater than or equal to the virtual degree of the new node. Thus, our previous model consists of the following two steps: the copying degree step and the OPA step. In the copying degree step, first an existing node *y* is chosen randomly. Then, the virtual degree $d_x^*$ of the new node *x* is given as a randomly chosen natural number *k* less than or equal to $\lceil \delta d_y \rceil$, where $d_y$ is the degree of *y* and $\lceil r \rceil$ is the smallest integer greater than or equal to a real number *r*. In the OPA step, $d_x^*$ existing nodes *z* satisfying $d_x^* \le \lceil \delta d_z \rceil$ are randomly chosen and the new node *x* forms links to them. When the number of existing nodes *z* satisfying the above inequality is less than $d_x^*$, *x* connects to all such nodes and the OPA step is completed.

Our previous model can generate dense scale-free networks with exponent $1<\gamma \le 2$ for $1 \le \delta < e=2.71828\dots$^11^. A theoretical analysis based on the rate equation shows that $\gamma$ is obtained as a non-trivial solution of $\gamma =\delta (\gamma -1)^2+\delta ^{\gamma -1}$. The range of power-law regime is given by $1 \ll k <Mt^{\frac{1}{\gamma }}$ for some constant $M>0$, where *t* is the number of nodes. The average degree diverges in proportional to $\ln t$ and $t^{\frac{2}{\gamma }-1}$ for $\gamma =2$ and $1< \gamma <2$, respectively. The degree correlation function $k_{\mathrm{nn}}(k)$ defined as the average degree of the neighbors of a node with degree *k* satisfies $k_{\mathrm{nn}}(k) \ge \frac{\delta }{2}k$ for $k \gg 1$, indicating a strong positive degree−degree correlation. For the local clustering coefficient *C*(*k*) defined as the probability that a pair of nodes among neighbors of a node of degree *k* is linked, we have $C(k) \ge \frac{D}{t}k^{\gamma }$ for $k \gg 1$ when $\delta <1.796028\dots$, where *D* is a constant depending on $\delta$. Thus, *C*(*k*) is expected to increase on average as *k* increases, which was verified by numerical simulation^11^.

## Results

We modify our previous model so that the modified model still can generate dense scale-free networks but with a different higher-order network structure. We show that the generated networks have an almost neutral degree−degree correlation and a negative scaling of local clustering coefficient. First, we analyze the duality structure of our previous model by category theory. Second, we synthesize a new model preserving the extracted duality structure.

**Figure 1:**
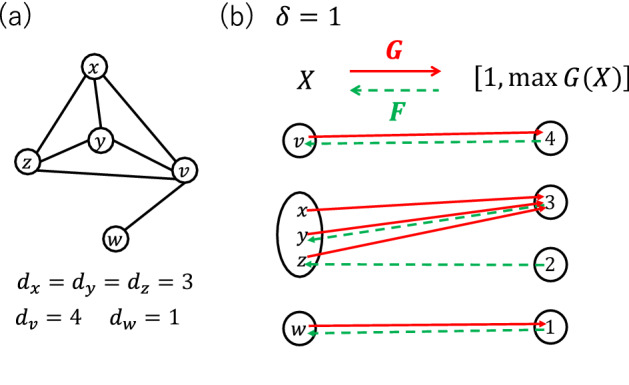
(**a**) A network consisting of five nodes *v*, *w*, *x*, *y* and *z*. (**b**) A Galois connection (*F*, *G*) associated with the network shown in (**a**). Here, we assume that $\delta =1$ and thus the map *G* is simply given by $G(x')=d_{x'}$ for all $x' \in X$. Equivalent elements in each preordered set are enclosed in the same round box. Even if we change the value of *F*(3) from *y* to *x* or *z*, (*F*, *G*) is still a Galois connection since *x*, *y* and *z* are equivalent.

### Analysis of the previous model

Let *X* be the set of nodes in a network generated by our previous model described in Section "Background". We define a map $G:X \rightarrow {\mathbb {N}}$ from *X* to the set of natural numbers ${\mathbb {N}}$ by $G(x)=\lceil \delta d_x\rceil$ for $x \in X$. Here, we assume that $d_x>0$ for all $x \in X$. This condition is satisfied in the course of growth if the initial network is a connected network with two or more nodes. We regard ${\mathbb {N}}$ as a preordered set by the usual less-than-or-equal-to relation $\le$ between natural numbers. We equip *X* with a preorder $\le _X$ by defining $x \le _X y :\Leftrightarrow d_x \le d_y$ for $x,y \in X$. The preorder $\le _X$ is a total preorder, namely, for any pair of nodes *x*, *y* in *X*, $x \le _X y$ or $y \le _X x$ hold. Two nodes $x,y \in X$ are equivalent, namely, both $x \le _X y$ and $y \le _X x$ hold, if and only if $d_x=d_y$. By this definition of $\le _X$, *G* becomes a preorder-preserving map. Namely, if $x \le _X y$ then $G(x) \le G(y)$ holds. Note that the converse implication also holds for $\delta \ge 1$, which corresponds to the dense regime of interest in this paper. Thus, $x \le _X y$ is equivalent to $G(x) \le G(y)$ when $\delta \ge 1$. *G* is the map evaluating the ability of each node to form links. The copying degree step in our previous model is restated as follows: Randomly choosing a natural number *k* such that $k \le G(y)$ for a randomly chosen node $y \in X$.

If we restrict the range of *G* to the interval $\left[ 1, \max G(X)\right] \subseteq {\mathbb {N}}$, then there exists a preorder-preserving map $F:\left[ 1, \max G(X)\right] \rightarrow X$ such that $F(k) \le _X x \Leftrightarrow k \le G(x)$ holds for all $x \in X$ and $k \in \left[ 1, \max G(X)\right]$. Concretely, *F*(*k*) is a minimum element of the set $\left\{ z \in X \mid k \le G(z) \right\}$, which is guaranteed to exist, since $\le _X$ is a total preorder and *X* is a finite set. Since *G* is a many-to-one map in general (see Fig. 1(b)), there can be multiple minimum elements. Any choice from the set of the minimum elements can be used to define *F*(*k*). The pair of preorder-preserving maps (*F*, *G*) is called a Galois connection, or an adjunction^16,18^. Figure 1(b) illustrates a Galois connection (*F*, *G*) for the network shown in Fig. 1(a). We can see that $F(G(x'))$ and $x'$ are equivalent for all $x' \in X$ in Fig. 1(b). This is true in general when $\delta \ge 1$.

**Figure 2:**
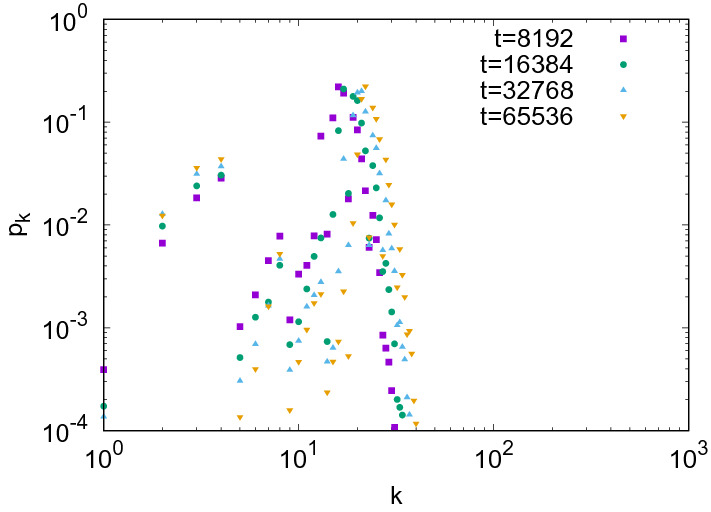
Evolution of the degree distribution of the generated networks by our previous model with the OPA step replaced by the adjunction rule for $\delta =\sqrt{\frac{3}{2}}$. The initial network consists of two different nodes and a link between them. The data are averaged over 100 trials.

Since *G* evaluates the ability of each node to form links, the dual map *F* to *G* can be regarded as expressing a ‘realization’ process of the ability to form links. Under this interpretation, it might be natural to introduce the following link formation rule: For $k \le G(y)$ obtained in the degree copying step, the targets of the links from the new node *x* with virtual degree $d_x^*=k$ is chosen as *F*(*k*). In more detail, let $M_k$ be the set of the minimum elements of $\left\{ z \in X \mid k \le G(z) \right\}$. If the size of $M_{d_x^*}$ is greater than or equal to $d_x^*$, then *x* connects to randomly chosen $d_x^*$ nodes in $M_{d_x^*}$. Otherwise, *x* connects to all nodes in $M_{d_x^*}$. We call this link formation rule the *adjunction rule*. However, the adjunction rule cannot produce dense scale-free networks as numerically demonstrated for $\delta =\sqrt{\frac{3}{2}}$ in Fig. 2. On the other hand, in the OPA step of our previous model, the targets of the links from the new node *x* are randomly chosen from the set $\left\{ z \in X \mid d_x^* \le G(z) \right\}$ rather than taking the minimum elements. We call this rule used in the OPA step the *pre-adjunction rule*.

### Synthesis of a new model

In Section "Analysis of the previous model", we express the duality of degree of nodes (result of link formation/cause of link formation) as a Galois connection (*F*, *G*). The copying degree step and the OPA step in our previous model are restated in terms of the Galois connection (*F*, *G*). In particular, it turns out that the OPA step uses the pre-adjunction rule for link formation. Now, we obtain a new growing network model by choosing a different but isomorphic Galois connection $(F',G')$ to (*F*, *G*).

First, we describe the algorithm of the new model without referring to category theory. Then, we explain how we obtain the new model from the Galois connection $(F',G')$.

Given an initial network, the following two steps are repeated indefinitely in the new model:

1. An existing node *y* is chosen uniformly at random from the set of all existing nodes. A new node *x* is generated with its virtual degree $d_x^*$. $d_x^*$ is a natural number chosen uniformly at random from the interval $[1,\lceil \delta d_y \rceil ]$, where $\delta >0$ is a parameter.
2. An existing node *z* is chosen uniformly at random from the set of all existing nodes $z'$ satisfying $d_x^* \le \lceil \delta d_{z'} \rceil$. Let $N_z$ be the union of the set of neighbors of *z* and $\{z\}$. If $d_x^*$ is less than the size of $N_z$, then *x* connects to $d_x^*$ nodes chosen uniformly at random from $N_z$. Otherwise, the targets of *x* are the all nodes of $N_z$.

Now, we explain the category theoretical derivation of the new model. We consider the set $N_X:=\left\{ (x,N_x) \mid x \in X \right\}$ instead of *X*. We define a map $G':N_X \rightarrow {\mathbb {N}}$ by $G'((x,N_x))=G(x)$. We introduce a preorder $\le _{N_X}$ on $N_X$ by $(x,N_x) \le _{N_X} (y,N_y) :\Leftrightarrow d_x \le d_y$ for $(x,N_x), (y,N_y) \in N_X$. Then, $G'$ is a preorder-preserving map. The preordered sets $(X,\le _X)$ and $(N_X,\le _{N_X})$ are isomorphic by the isomorphism $I:X \rightarrow N_X$ defined by $I(x)=(x,N_x)$ for each $x \in X$ and we have $G'=G \circ I^{-1}$. Thus, the preorder-preserving maps $F':=I \circ F :\left[ 1, \max G'(N_X)\right] \rightarrow N_X$ and $G'$ form a Galois connection $(F',G')$. For $k \in \left[ 1, \max G'(N_X)\right]$, $F'(k)$ is a minimum element of the set $\left\{ (x,N_x) \in N_X \mid k \le G'((x,N_x)) \right\}$.

Based on the pre-adjunction rule for $(F',G')$, we obtain the above new growing network model consisting of the copying degree step and a new version of the OPA step. We adopt the same procedure for the copying degree step. Let *x* be a new node to be added and $d_x^*$ its virtual degree. According to the pre-adjunction rule for $(F',G')$, the targets of *x* is chosen from the set $\left\{ (z,N_z) \in N_X \mid d_x^* \le G'((z,N_z)) \right\}$. However, since $(z,N_z)$ is not a node but a pair of a node and a set of nodes, there is an arbitrariness how they are chosen. Here, we adopt the following procedure: $d_x^*$ nodes are chosen randomly from $N_z$, where $(z,N_z)$ is chosen randomly from the above set. If the size of chosen $N_z$ is less than $d_x^*$, *x* connects to all nodes in $N_z$ and complete the OPA step. In short, the new node *x* can make a new link with existing nodes *z* whose ability to form links is greater than or equal to that of *x* and *z*’s neighbors.

We expect that this link formation rule has following effects to degree−degree correlation and local clustering. Since a *z*’s neighbor $z'$ does not necessarily satisfy $d_x^* \le G'((z',N_{z'}))$, degree−degree correlation can be weakened compared to that in our previous model where the inequality is satisfied for all targets of *x*. On the other hand, if the set of chosen targets of *x* includes both *z* and its neighbors $z'$, triangles among *x*, *z*, and $z'$ are formed, increasing the degree of local clustering. This mechanism can result in a negative scaling of local clustering coefficient^29^.

**Figure 3:**
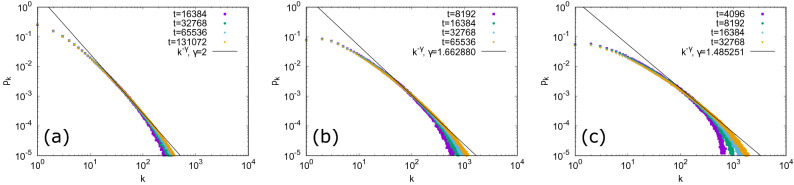
Evolution of the degree distribution of the generated networks by the proposed model. (**a**) $\delta =1$, (**b**) $\delta =\sqrt{\frac{3}{2}}$, and (**c**) $\delta =\sqrt{2}$. The values of the power-law exponent are given by Eq. (1). The data are averaged over 100 trials.

**Figure 4:**
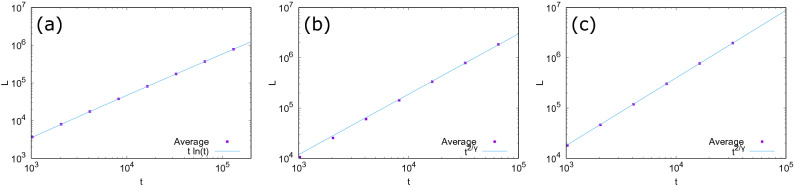
The average number of links. (**a**) $\delta =1$, (**b**) $\delta =\sqrt{\frac{3}{2}}$, and (**c**) $\delta =\sqrt{2}$. Solid lines are the best fitting of the right-hand-side of Eq. (2). The data are averaged over 100 trials.

**Figure 5:**
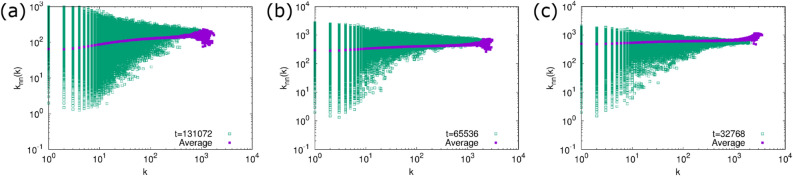
The degree correlation function $k_{\mathrm {nn}}(k)$ of the generated networks at the final time step in the numerical simulation. (**a**) $\delta =1$, (**b**) $\delta =\sqrt{\frac{3}{2}}$, and (**c**) $\delta =\sqrt{2}$. Open squares are the result for a single trial and filled squares are the average over 100 trials.

**Figure 6:**
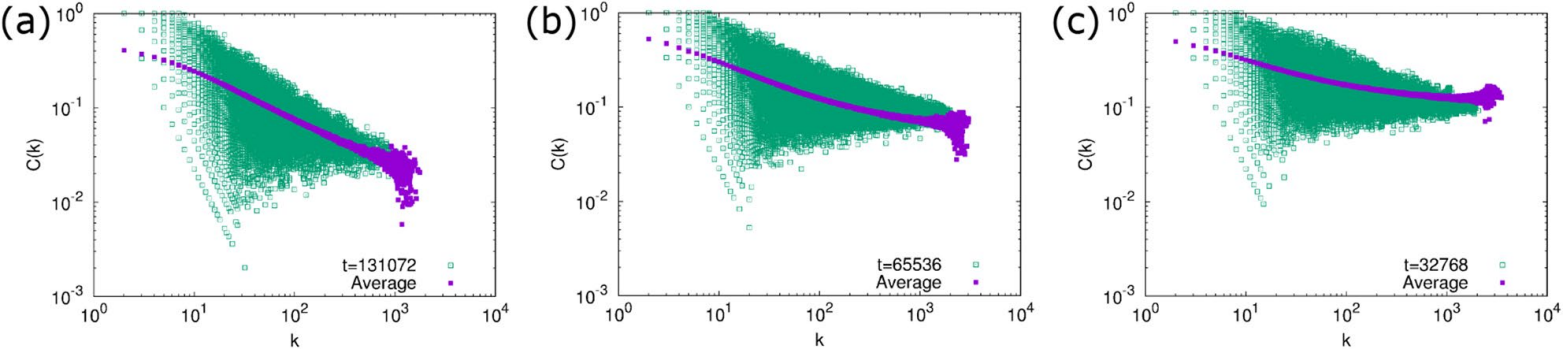
The local clustering coefficient *C*(*k*) of the generated networks at the final time step in the numerical simulation. (**a**) $\delta =1$, (**b**) $\delta =\sqrt{\frac{3}{2}}$, and (**c**) $\delta =\sqrt{2}$. Open squares are the result for a single trial and filled squares are the average over 100 trials.

The new model can generate dense scale-free networks when $1 \le \delta <e$. In the following, we focus on this parameter range. An analysis based on the rate equation^30^ similar to that in our previous work^11^ shows that if we assume that $p_k \sim k^{-\gamma }$ for $1 \ll k < M't^{\frac{1}{\gamma }}$, where $1<\gamma \le 2$, $M'>0$ is a constant, and the upper limit $M't^{\frac{1}{\gamma }}$ comes from the constraint on the allowed maximum degree for dense scale-free networks^5^, and that the degree of a neighbor of a randomly chosen node is independent of the degree of the chosen node, then we self-consistently obtain (see "Methods")

1 $$\begin{aligned} \gamma =1+\frac{1-\ln \delta }{1+\ln \delta }. \end{aligned}$$

In Fig. 3, degree distributions of numerically simulated networks for three different values of $\delta$ ($\delta =1,\sqrt{\frac{3}{2}},\delta =\sqrt{2}$) are compared with the theoretical prediction. Here, *t* denotes the number of nodes and the initial condition is given as the network with two different nodes and a single link between them. As networks grow, the scale-free regime is enlarged and the slope in the log-log plot agrees with the value obtained from Eq. (1). The number of links *L* scales as

2 $$\begin{aligned} L=\frac{t}{2} \langle k \rangle \sim t \int ^{M't^{\frac{1}{\gamma }}} dk \, k^{-\gamma +1} \sim \left\{ \begin{array}{ll} t \ln t &{} (\gamma =2) \\ t^{2/\gamma } &{} (1<\gamma <2), \end{array} \right. \end{aligned}$$

where $\langle k \rangle$ is the average degree. In other words, the average degree $\langle k \rangle$ diverges as $\ln t$ and $t^{2/\gamma -1}$ for $\delta =1$ and $1<\delta <e$, respectively. Thus, the generated networks are expected to be dense. Indeed, Fig. 4 compares the result of numerical simulation and Eq. (2) for the number of links *L*, showing that they are consistent.

The numerical results for the degree correlation function $k_{\mathrm{nn}}(k)$ and the local clustering coefficient *C*(*k*) are shown in Figs. 5 and 6, respectively. The average of $k_{\mathrm{nn}}(k)$ is almost constant and thus is consistent with the assumption of the above rate equation analysis. *C*(*k*) tends to decrease as *k* increases, which is the opposite trend against *C*(*k*) of the generated networks by our previous model. The behavior of $k_{\mathrm{nn}}(k)$ and *C*(*k*) is consistent with our expactation from the link formation rule discussed above.

## Discussion

In this paper, we apply category theory for building a new growing network model that can generate dense scale-free networks. The proposed model is constructed through a modification of our previous model while preserving the duality associated with it. Both our previous and proposed models can generate dense scale-free networks. However, their higher-order network structures are different: Those generated by the former have a positive degree−degree correlation and a positive scaling of local clustering coefficient, while those generated by the latter have an almost neutral degree−degree correlation and a negative scaling of local clustering coefficient.

In Section "Analysis of the previous model", we have observed that the adjunction rule for link formation does not work for generating power-law degree distributions. In the pre-adjunction rule adopted in our previous and proposed models, a kind of fluctuation is introduced, which is crucial for generating dense scale-free networks: Taking minimum elements of a set is replaced by a random choice from the set. Formally, such incorporation of randomness can be readily extended to category theoretical limits or colimits, which are generalizations of minimum or maximum elements of a subset of a preordered set^16,17^. Studying whether such extension of the pre-adjunction rule is meaningful or not in different mathematical models will be investigated elsewhere.

The category theoretic duality of nodes’ degree described in Section "Results" is mathematically rather trivial. However, we have shown that it guides construction of a non-trivial mathematical model of growing networks. In one of the authors’ previous work^31,32^, category theory was applied for analyzing the structure of static networks. In this paper, we have presented a novel kind of application of category theory, namely, designing of a dynamic network model. We hope that such an extended application of category theory leads to deepening understanding of mathematical structures of models for networks.

## Methods

### Preordered sets and Galois connections

For a reference on the material in this section, we refer to Chapter 1 of Fong and Spivak^18^.

Let *X* be a set. A *preorder* on *X* is a binary relation $\le _X \subseteq X \times X$ satisfying the following two conditions: (i) $x \le _X x$ for all $x \in X$ (reflexivity), and (ii) if $x \le _X y$ and $y \le _X z$, then $x \le _X z$ for all $x,y,z \in X$ (transitivity). A set *X* equipped with a preorder $\le _X$ is called a *preordered set* and is denoted by $(X,\le _X)$. Two elements $x,y \in X$ are called *equivalent* when both $x \le _X y$ and $y \le _X x$ hold. The preorder $\le _X$ is called a *total preorder* when $x \le _X y$ or $y \le _X x$ hold for all $x, y \in X$.

Let $(X,\le _X)$ and $(Y,\le _Y)$ be preordered sets. A map $F:X \rightarrow Y$ is called a *preorder-preserving map* when *F* preserves the preorder, namely, it holds that if $x_1 \le _X x_2$ then $F(x_1) \le _Y F(x_2)$ for all $x_1,x_2 \in X$. Let $F: X \rightarrow Y$ be a preorder-preserving map. If there exists a preorder-preserving map $G: Y \rightarrow X$ satisfying $G \circ F=\mathrm {id}_X$ and $F \circ G=\mathrm {id}_Y$, where $\mathrm {id}_X$ and $\mathrm {id}_Y$ are the identity maps on *X* and *Y*, respectively, then *F* is called an *isomorphism* between $(X,\le _X)$ and $(Y,\le _Y)$. Here, *G* is also an isomorphism and is denoted by $G=F^{-1}$.

Let $F: X \rightarrow Y$ and $G: Y \rightarrow X$ be preorder-preserving maps for preordered sets $(X,\le _X)$ and $(Y,\le _Y)$. A pair of preorder-preserving maps (*F*, *G*) is called a *Galois connection* or an *adjunction* between $(X,\le _X)$ and $(Y,\le _Y)$ if $F(x) \le _Y y \Leftrightarrow x \le _X G(y)$ holds for all $x \in X$ and $y \in Y$.

Let $(X,\le _X)$ be a preordered set and $Z \subseteq X$ a subset. $z^* \in Z$ is called a *minimum element* of *Z*, when $z^* \le _X z$ for all $z \in Z$.

Let (*F*, *G*) be a Galois connection as above. It holds that, for each $x \in X$, *F*(*x*) is a minimum element of $Z:=\left\{ y \in Y \mid x \le _X G(y) \right\} \subseteq Y$. Indeed, since $F(x) \le _Y F(x)$, we have $x \le _X G(F(x))$, which shows $F(x) \in Z$. We also have, for any $y \in Z$, $x \le _X G(y)$, which is equivalent to $F(x) \le _Y y$. Thus, *F*(*x*) is a minimum element of *Z*.

### Derivation of Eq. (1)

Let $1 \le \delta < e$. Let $p_k(t)$ be the fraction of nodes of degree *k* when the number of existing nodes in a network generated by the proposed model is equal to *t*. In the following, we assume $k>0$ since we are interested in the regime $k \gg 1$.

The time evolution of $p_k(t)$ follows the rate equation

3 $$\begin{aligned} (t+1)p_k(t+1) = t p_k(t) + a_{k-1}(t) t p_{k-1}(t) - a_k(t) t p_k(t) + b_k(t), \end{aligned}$$

where $a_k(t)$ is the probability that an existing node of degree *k* gets a link from a new node *x* when the number of existing nodes is *t*, and $b_k(t)$ is the probability that the node newly added has degree *k*. Let $q_k(t)$ be the probability that $d_x^*=k$. We have

4 $$\begin{aligned} q_k(t)=\sum _{k \le \lceil \delta l \rceil < t} p_l(t) \times \frac{1}{\lceil \delta l \rceil }, \end{aligned}$$

and

5 $$\begin{aligned} b_k(t) = q_k(t) \times \frac{ \sum _{k \le l< t} p_l(t) }{ \sum _{k \le \lceil \delta l \rceil< t} p_l(t) } + \sum _{k< l \le \lceil \delta k \rceil } q_l(t) \times \frac{ p_k(t) }{ \sum _{l \le \lceil \delta m \rceil < t} p_m(t) }. \end{aligned}$$

Let $N_k(t):=t \sum _{k \le \lceil \delta l \rceil < t} p_l(t)$. Let $d_k(t)$ be the probability that $N_z$ for a specific node *z* of degree *k* is chosen and a specific node in $N_z$ is chosen as the target for a link from the new node *x*. $d_k(t)$ is given by

6 $$\begin{aligned} d_k(t) = \sum _{l \le k} q_l(t) \times \frac{1}{N_l(t)} \times \frac{l}{k+1} + \sum _{k < l \le \lceil \delta k \rceil } q_l(t) \times \frac{1}{N_l(t)} \end{aligned}$$

Let us assume that the degree of a neighbor of a randomly chosen node is independent of the degree of the latter node. Then, the probability that the former is of degree $k'$ given the latter is of degree *k*, denoted by $p(k' | k)$, does not depend on *k* and is given by $p(k' | k)=\frac{k'p_{k'}(t)}{\langle k' \rangle }$. Using this, we obtain

7 $$\begin{aligned} a_k(t) = d_k(t) + k \sum _{k'} p(k' | k) \times d_{k'}(t). \end{aligned}$$

Now, we assume that $p_k(t) \simeq c k^{-\gamma }$ for $1 \ll k <Mt^{1/\gamma }$ and $t \gg 1$, where $c,M>0$ are appropriate constants and $1<\gamma \le 2$. We have

8 $$\begin{aligned} q_k(t) \simeq \int _{k/\delta }^\infty dl \, \frac{c}{\delta } l^{-\gamma -1} = \frac{c\delta ^{\gamma -1}}{\gamma } k^{-\gamma }. \end{aligned}$$

Similarly,

9 $$\begin{aligned}&b_k(t) \simeq \frac{c \left( 1+(\gamma -1)\ln \delta \right) }{\gamma } k^{-\gamma }, \end{aligned}$$

10 $$\begin{aligned}&d_k(t) \simeq \frac{\left( \gamma - 1 \right) \left( 1+\ln \delta \right) }{\gamma t}, \end{aligned}$$

and

11 $$\begin{aligned} a_k(t) \simeq \frac{\left( \gamma - 1 \right) \left( 1+\ln \delta \right) (k+1)}{\gamma t} \simeq \frac{\left( \gamma - 1 \right) \left( 1+\ln \delta \right) k}{\gamma t}, \end{aligned}$$

for $1 \ll k <Mt^{1/\gamma }$ and $t \gg 1$. Plugging $p_k(t) \simeq c k^{-\gamma }$ and Eqs. (9) and (11) into the continuous approximation of Eq. (3) with respect to *k* for $t \gg 1$

12 $$\begin{aligned} \frac{\partial }{\partial k}\left( a_k(t) t p_k(t)\right) = b_k(t) -p_k(t), \end{aligned}$$

and comparing the coefficients of $k^{-\gamma }$ on the both sides of Eq. (12), we obtain Eq. (1):

13 $$\begin{aligned} \gamma =1+\frac{1-\ln \delta }{1+\ln \delta }. \end{aligned}$$

## Data Availability

The codes and the data that support our findings in this paper are available on the GitHub https://github.com/taichiharuna/opa2.
